# Immunosenescence and human vaccine immune responses

**DOI:** 10.1186/s12979-019-0164-9

**Published:** 2019-09-13

**Authors:** Stephen N. Crooke, Inna G. Ovsyannikova, Gregory A. Poland, Richard B. Kennedy

**Affiliations:** Mayo Clinic Vaccine Research Group, Mayo Clinic, Guggenheim Building 611D, 200 First Street SW, Rochester, MN 55905 USA

**Keywords:** Immunosenescence, Vaccination, Aging, Immune response, T cell, B cell, Adaptive immunity

## Abstract

The age-related dysregulation and decline of the immune system—collectively termed “immunosenescence”—has been generally associated with an increased susceptibility to infectious pathogens and poor vaccine responses in older adults. While numerous studies have reported on the clinical outcomes of infected or vaccinated individuals, our understanding of the mechanisms governing the onset of immunosenescence and its effects on adaptive immunity remains incomplete. Age-dependent differences in T and B lymphocyte populations and functions have been well-defined, yet studies that demonstrate direct associations between immune cell function and clinical outcomes in older individuals are lacking. Despite these knowledge gaps, research has progressed in the development of vaccine and adjuvant formulations tailored for older adults in order to boost protective immunity and overcome immunosenescence. In this review, we will discuss the development of vaccines for older adults in light of our current understanding—or lack thereof—of the aging immune system. We highlight the functional changes that are known to occur in the adaptive immune system with age, followed by a discussion of current, clinically relevant pathogens that disproportionately affect older adults and are the central focus of vaccine research efforts for the aging population. We conclude with an outlook on personalized vaccine development for older adults and areas in need of further study in order to improve our fundamental understanding of adaptive immunosenescence.

## Introduction

**Fig. 1:**
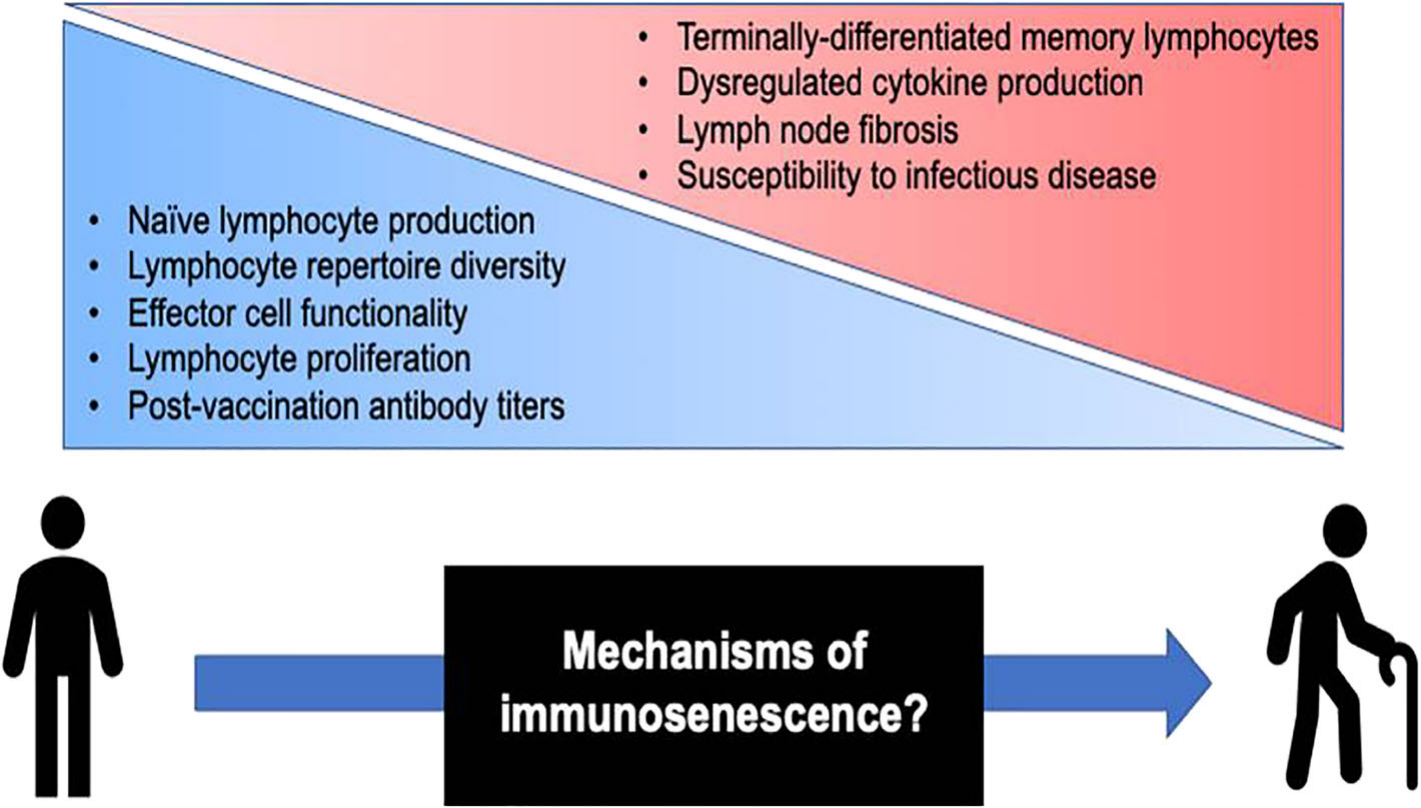
Immunological changes associated with aging and adaptive immunosenescence. Specific changes in the T and B cell compartments are known to occur with aging and the onset of immunosenescence. Naïve lymphocyte production, lymphocyte repertoire diversity, and the proliferative and functional capacity of effector lymphocytes all decline with age; similarly, increases in differentiated memory cell populations, lymph node fibrosis, and altered cytokine production all occur. These phenomena have been collectively associated with diminished vaccine responses and an increased susceptibility to viral infectious diseases in older adults. The mechanisms by which immunosenescence operates are not fully understood, and systems biology approaches are currently focused on elucidating these mechanisms in order to inform the rational design of vaccines for older adults

Aging is associated with the decline of various biological systems and the development of numerous co-morbidities, such as diabetes mellitus, cancer, and various autoimmune and neurological disorders [1]. The immune system suffers equally from the effects of biological aging, exhibiting a progressive decline in function—referred to as immunosenescence—that collectively results in diminished humoral and cellular immune responses [2–4]. (Figure 1) Mechanistic analyses of immunosenescence are complicated by the integrated nature of the immune system, as it is difficult to discern if immune cell dysregulation is a result of inherent cellular changes or a reactionary mechanism to changes elsewhere in the body. Regardless of origin, functional differences in the aging immune system are well-documented [5, 6], and several studies have attributed negative clinical outcomes in populations of older adults (i.e., 65 years and older) to immunosenescence. The severity of viral and bacterial infections (e.g., influenza, respiratory syncytial virus [RSV], herpes zoster, pneumococcal disease) is notably increased among older adults compared to younger individuals, and more acute and long-term sequelae often develop as a result [7–10]. Vaccination serves as the main strategy for preventing such infections, yet primary vaccine responses are often lower in older adults, frequently failing to induce long-term protective immunity and placing these individuals at further risk for subsequent disease [11–14]. These findings have been predominantly linked to the function and perceived failure of the adaptive immune response in older adults.

Vaccination has been the crowning achievement of modern preventive medicine, establishing protective immunity among the population to prevent and, in some cases, eradicate infectious disease [15]. Durable protective immunity is mediated by the adaptive immune system through the induction of antigen-specific T cell and B cell responses, which establish immunological memory against offending pathogens and prevent subsequent infections. The success of vaccination among older adults is decidedly limited, and adaptive immunosenescence has been implicated as a key determinant by numerous studies investigating vaccine responses in aging populations. Diminished antibody titers have been observed among older adults [16, 17], and, in many cases, the quality of these antibody responses is markedly inferior compared to those in their younger counterparts [18–20]. It should be noted that declining antibody quality is not a universal age-associated phenomenon, as studies evaluating influenza and tick-borne encephalitis virus vaccines have found no differences in antigen-specific antibody affinity or avidity between age groups [21, 22]. The T cell compartment is also affected during aging, exhibiting contraction of the naïve T cell repertoire [23] and accumulation of terminally differentiated cell subsets with altered effector functions [24, 25]. Additionally, an underappreciated restructuring of lymph node architecture occurs with aging [26, 27], which could further alter critical processes in the development of adaptive immune responses. Despite these observations of age-related changes in adaptive cell function, few studies have definitively shown that these age-related phenomena are causal to diminished immune responses against infection or vaccination.

The need for improved clinical outcomes among older adults has driven research efforts in vaccine and adjuvant design despite our limited mechanistic understanding of immunosenescence or its effects on adaptive immunity. Several vaccine formulations have already been developed and licensed for diseases that disproportionately affect older adults, including influenza [28, 29] and herpes zoster (i.e., shingles) [30], and several others are currently undergoing preclinical and clinical testing. Many of these vaccines have resulted in significantly improved immune responses, yet more research is needed to determine the mechanisms by which these vaccines act to bolster the immune response. As the field of vaccinology races to keep pace with the aging global population, it is imperative that we fully understand the mechanistic changes that occur in the immune system during aging in order to develop effective vaccines that can overcome these changes.

In this review, we outline the changes that occur in the adaptive immune system during aging—specifically in T and B cells—with a focus toward understanding the mechanisms of immunosenescence that might govern clinical outcomes following vaccination. Although the contributions of innate immunity to adaptive immune responses have become more appreciated in recent years [31], we limit our discussion to age-related changes in the adaptive immune system, as the effects of immunosenescence on innate immunity and advancements in adjuvant research to overcome these changes have been reviewed elsewhere [32–35]. This review is largely focused on diseases that are major public health concerns for older adults, and we also highlight how studies of adaptive immunosenescence can guide vaccine research efforts in this population. We also provide an outlook on personalized vaccine development (i.e., vaccines developed specifically for older adults), emphasizing the gaps that remain in our current understanding of the aging immune system and highlighting promising approaches to further vaccine research efforts in support of healthy aging.

## Dysregulation and decline of adaptive immunity with aging

Our current understanding of immunosenescence implicates changes in the adaptive immune system—particularly within T cell and B cell populations—as the primary determinants of declining immune function with age. While compelling evidence has been presented toward understanding the mechanisms governing adaptive immunosenescence, one must carefully consider the nature of these studies when interpreting their clinical relevance. Herein, we provide an overview of the changes that have been observed in the adaptive immune system during aging, with a focus on alterations that might impact immune responses to infection or vaccination. While we appreciate that a multitude of anatomical and physiological changes occur during aging that may also increase the risk of infection, a discussion of these are beyond the scope of this review. These topics have been reviewed elsewhere [36–39].

### Alterations in cellular immunity

While the overall number of T cells in circulation remains relatively constant during life, multifaceted changes are known to occur among the T cell populations of older adults; these alterations have been largely implicated in the decline of adaptive immune responses with aging. Perhaps the most striking change that occurs within the aging T cell compartment is the diminished output of new naïve T cells (e.g., CD45RA^+^CD45RO^−^CD62L^+^CCR7^+^) as a result of thymic involution [40]. The thymus, which is the specialized lymphoid organ where thymocytes develop into mature naïve T cells, begins to functionally decline as early as the first year of life, and this process definitively accelerates following the onset of puberty [41]. This decline is thought to originate with defects in the stroma, which is consistent with histological analyses that show progressive disorganization of epithelial cell structure in the thymus with age [5]. Defects in the production of cytokines and growth factors by thymic epithelial cells have also been implicated as causal mediators of declining thymic function [5, 42]. As a result, the number of naïve T cells entering the periphery declines proportionally with age, as evidenced by fewer cells expressing markers of recent thymic emigration (e.g., CD31^+^ for CD4^+^ T cells/CD103^+^ for CD8^+^ T cells) [43].

Maintenance of the naïve T cell repertoire in humans has been found to be notably independent of thymic output, with the decline in thymic productivity counterbalanced by homeostatic proliferation of existing naïve T cells in the periphery [44]. This process relies upon subthreshold T cell receptor (TCR) stimulation or low-grade cytokine signaling necessary for cellular survival (IL-7) and maintenance (IL-15) [45], and is generally more effective at maintaining the CD4^+^ T cell compartment compared to CD8^+^ T cells [46]. While the reason for this discrepancy is unknown, it is generally well-accepted that CD8^+^ T cells undergo higher rates of turnover and longer rounds of proliferation compared to CD4^+^ T cells [47, 48]. These processes result in depletion of the naïve CD8^+^ pool through either increased rates of apoptosis or differentiation into “virtual” memory T cells (T_VM_; CD44^hi^CD122^hi^CD49d^lo^ in mice and CD45RA^+^PanKIR^+^ and/or NKG2A^+^ in humans), which are antigen-specific despite no prior antigenic encounters [25, 49–51]. T_VM_ cells from both mice and humans exhibit reduced proliferative capacity despite maintaining the ability to secrete cytokines, although their prolonged life cycles lead to gradual crowding of the T cell compartment [25]. Eventually, homeostatic proliferation succumbs to age-related dysregulation and results in the increased differentiation of naïve T cells from both compartments into T_VM_-like cells [50, 52, 53]. This gradual decline in the production of new T cells, combined with the accumulation of terminally differentiated memory-like cells in the periphery, contributes to an overall contraction of the naïve T cell repertoire and limits the ability of the immune system to effectively respond to encounters with novel antigens.

Prior antigen exposure can significantly alter TCR diversity among the expanded pool of aged memory T cells, particularly in the case of chronic infections that can repeatedly antagonize the immune system throughout life. Cytomegalovirus (CMV) is a herpesvirus that commonly infects 60–90% of older adults, and persistent infection with CMV has been associated with deficits in cellular immune responses and increased health risks with aging [54, 55]. Repeated response to CMV infection leads to inflation of the memory compartment with CMV-specific clones and may place considerable limitations on the responsiveness of the CD8^+^ repertoire toward other antigens, as this oligoclonal expansion minimizes the space and resources necessary to maintain T cells with other specificities [56]. Studies in mice have found that immune responses against novel antigens are significantly suppressed by existing CMV infection that correlates with impaired mobilization of naïve CD8^+^ T cells into the lymph nodes [57], and negative associations have also been made between CMV seropositivity and vaccine responses in older adults [58]. The mechanistic effects of CMV infection on humoral immune responses are far less understood [59, 60], and the lower antibody responses associated with CMV infections may likely be a result of compromised CD4^+^ T cell activity. Interestingly, one study did note a significant overall decline of naïve CD4^+^ T cells in CMV-infected individuals [46]. Furthermore, CMV-specific T cells develop a phenotype consistent with replicative senescence (e.g., CD45RA^+^CD57^+^CD28^−^CCR7^−^) and exhibit limited cytokine expression in response to antigenic stimuli [24, 61]. It is clear that memory repertoire responses are negatively impacted by persistent CMV infection, although the mechanisms governing these phenomena have yet to be elucidated.

Dysregulation within the T cell compartment during aging extends well beyond changes in cell numbers and shifts in population frequencies; fundamental alterations in cellular effector functions also play a key role. Memory T cells from older adults generally exhibit diminished proliferative capacity and produce lower levels of cytokines in response to antigenic challenges. The age-associated decline in expression of the costimulatory receptor CD28 has been correlated with decreased cellular proliferation and incomplete T cell activation following influenza vaccination in older adults [62–64], and responsiveness to TCR signaling has also been shown to be attenuated in aged CD4^+^ and CD8^+^ memory T cells [65, 66]. Cytokine responses are also highly dysregulated among the T cell compartment with increasing age, with a shift toward a Th2-type cytokine profile that may explain some of the observed deficiencies in Th1-type responses among older adults [67]. Effector memory cell subsets from aged mice and humans have also shown diminished cytokine production in response to antigen stimulation [68, 69], while terminally differentiated senescent CD4^+^ T cells exhibit increased production of proinflammatory cytokines in aged mice and may potentially contribute to the state of chronic inflammation that is commonly observed with aging [70]. Collectively, these changes in T cell function demonstrate a diminished ability to effectively respond to antigens along with shifts in cytokine production that may further contribute to age-related immune dysfunction.

Dendritic cells (DCs) serve as the bridge between innate and adaptive immunity by presenting antigenic and “self” peptides and costimulatory signals to T cells in the draining lymph nodes. While the age-associated changes in DC function have been reviewed elsewhere [71, 72], it is important to consider those changes that may impact DC-T cell cross-talk and the establishment of robust adaptive immune responses. Circulating DC numbers remain approximately stable with age, with only plasmacytoid DCs (pDCs) exhibiting significant declines in number [73, 74]; however, cytokine-secretion profiles differ significantly among all DC subsets. pDCs from older adults exhibit a marked decrease in IFN secretion in response to influenza and other stimuli [73–76], and the production of pro-inflammatory cytokines (e.g., IL-6, TNF-α, IL-12p40) in response to a variety of Toll-like receptor (TLR) agonists is significantly decreased in myeloid DCs (mDCs) from older adults [73]. Further studies are required to elucidate if dysregulation in cytokine signaling among DCs is causally associated with declining T cell responses in older adults, as several DC subsets—particularly tissue-resident subsets—have not been thoroughly investigated.

Changes in the expression levels of co-stimulatory molecules on aging DCs are even less well-defined. Human monocyte-derived DCs (MoDCs) from older adults maintain the ability to upregulate HLA class I and II molecules, as well as co-stimulatory proteins (e.g., CD40, CD80, CD86), in response to TLR stimulation [77, 78], whereas these responses are notably diminished in aged murine DCs responding to infection [79, 80]. Studies in humans have been primarily limited to MoDCs, while the expression of co-stimulatory molecules in DC subsets from the circulation and other tissues remains poorly understood. Despite maintaining their ability to express stimulatory molecules, current evidence suggests that MoDCs from older adults are less efficient at stimulating naïve CD4^+^ and CD8^+^ T cell proliferation and cytokine responses compared to MoDCs from younger subjects [69, 75, 81]. This has been attributed to the state of chronic inflammation observed among older adults, which could disrupt DC signaling and inhibit T cell activation. Clearly, further studies are warranted to fully understand the functional changes occurring in DC subsets with age and address the discrepancies observed between human and murine DC function.

### Alterations in humoral immunity

While changes within the T cell compartment and DC subsets certainly have the potential to impact the development of humoral immune responses, there have also been several observations of age-related defects occurring directly within the B cell compartment. Interestingly, these changes appear to closely mirror those affecting T cells. Analogous to T cell output in the wake of thymic involution, the production of naïve B cells (e.g., IgD^+^IgM^+^CD19^+^CD27^−^) in mice has also been shown to decline due to age-related changes to the bone marrow (BM) [82, 83]. Studies in aged mice have identified chronic inflammation in the BM microenvironment—as well as decreased IL-7 production by BM stromal cells—as correlates of poor fitness for B cell progenitors [84, 85]. Additionally, intrinsic changes to hematopoietic stem cells within the BM result in developmental biases that exclude commitment to the lymphoid lineage [86, 87]. While analogous studies in humans are limited, numerous reports have noted a similar decline in naïve B cell numbers among older adults [82, 88]. Interestingly, decreased serum levels of B-cell activating factor (BAFF) and a proliferation-inducing ligand (APRIL), which are critical survival factors for B cells, have been correlated with poor B cell survival in older individuals [89]. Regardless of mechanism, replenishment of the circulating B cell repertoire with new B cell clones is significantly diminished with age in both mouse and human studies, impairing one’s ability to recognize and respond to new antigens.

Dysregulation of B cell homeostasis has also been found to have a noted impact on the composition and functionality of mature B cells in the periphery. As with the T cell compartment, there is a progressive accumulation of peripheral memory cells (e.g., IgD^−^CD19^+^CD27^+^) with age [82, 90, 91], although there are conflicting reports showing that these numbers remain unchanged in some cases [88]. A selective shift toward the production of IgG/IgA, along with observations of diminished antibody responses against novel antigens, suggests that the periphery does become inflated with class-switched memory B cells during aging [92]. This increase in the memory B cell population, along with the putative decrease in naïve B cell output from the BM, results in an overall contraction of the B cell repertoire that limits the number of clones available to respond to new antigens. In addition to these changes in repertoire composition, B cells also experience inherent functional defects with age that compromise their ability to effectively respond to antigens. Aged memory B cells exhibit a diminished capacity for differentiation into plasma cells post-challenge [93], and antigen-specific antibody production has been subsequently noted to decline with age [94]. Concurrently, an increase in the percentage of late memory B cells (e.g., IgD^−^CD95^hi^CD27^−^) spontaneously secreting TNF-α has been identified in elderly individuals, with this contribution to the inflammatory state implicated in further dysregulation of immune homeostasis and B cell function [95, 96]. Collectively, these changes to B cell subset populations and their functional capacity lead to diminished antibody production, with the quality of these antibody responses directly impacted by inherent changes within B cells.

Protective humoral immune responses are dependent upon the generation of isotype-switched, high-affinity antibodies within germinal centers, yet intrinsic defects in the mechanisms for class-switch recombination (CSR) and somatic hypermutation (SHM) adversely impact the quality of these responses with age. Studies have found that CSR and the production of effector isotypes are significantly impaired in senescent mice, and these outcomes are directly associated with reduced expression of activation-induced cytidine deaminase (AID) [97, 98]. AID is essential for CSR and SHM, and its expression is directly regulated by the transcription factor E47 [99]. The stability of E47 mRNA transcripts is significantly reduced in aged B cells from both humans and mice, which results in reduced E47 protein production and a subsequent decline in AID expression [100–102]. AID activity has also been shown to correlate with antibody affinity following influenza vaccination [103], further indicating the importance of these transcriptional mechanisms for proper B cell function.

The diversity of the B cell repertoire has also been found to significantly diminish with age, at least partially as a result of declining naïve B cell output combined with reduced germinal center activity [104, 105]. V(D) J recombination during initial B cell maturation introduces diverse changes within the third complementarity-determining region (CDR3) of immunoglobulin binding sites [106]. Spectratype analysis of the CDR3 region in older adults has indicated that a collapse of B cell diversity along with oligoclonal expansion within the repertoire occurs, and these phenomena have been shown to correlate with poor health outcomes [91, 107]. Interestingly, significant increases in the size of the CDR-H3 region for both IgA and IgM isotypes have been observed in older individuals following influenza vaccination, with these changes to the CDR3 region altering the antigen-binding sites and presumably diminishing antigen recognition [108]. Age-associated changes in follicular dendritic cells (FDCs) have also been shown to impact the development of robust humoral responses and the maturation of high-affinity antibodies due to the critical role that FDCs play in germinal center reactions [109]. Antigen trapping and presentation are compromised in FDCs from aged mice due to reduced expression of Fc receptors, which severely limits the number and size of germinal centers that develop in the lymph nodes [110].

### Alterations in lymph node architecture

While the discussion thus far has focused largely on inherent changes in cellular mechanisms, it is worth noting that architectural changes within lymphoid tissues— specifically, lymph nodes (LNs)—may play a previously underappreciated role in shaping immune responses with age. LN size has been shown to decline with age [26], and decreased LN swelling has also been noted in older mice following subcutaneous viral infection, indicative of a reduced influx of immune cells to the LN [111, 112]. Structural collapse has also been observed within the LNs of aged mice, with T cell and B cell zone boundaries becoming less well-defined [27]. Signs of fibrosis have been noted in aging LNs, and these architectural shifts have the potential to impair both lymphocyte homeostasis and the migration of immune cells during the course of an immune response [26, 112]. Fibroblastic reticular cells (FRCs) comprise a large portion of the LN stromal network, creating channels for chemokine transport and promoting DC and T cell migration [113]. The number of FRCs has been noted to decline with age, resulting in subsequent disorganization of this transport network in the LN [27]. Similar degrees of fibrosis and disorganization in the LN have been observed in the context of human immunodeficiency virus (HIV) and other viral infections, and reduced yellow fever vaccine responses have been correlated with levels of fibrosis and T cell depletion in the LNs [114]. As with many other aspects of aging, these changes within the LNs and their impact on immune response outcomes are an area that warrants more intensive study. This is also further evidence that immunosenescence is the net effect of an integrated network of changes occurring throughout the body and across multiple cell types. This complexity has hampered our ability to fully understand immunosenescence and its effects on vaccine immune responses.

## Senescence-associated pathologies

Changes to the adaptive immune system with age have a clear impact on immune cell function, and it is well-documented that older adults are more susceptible to infections and suffer more long-term complications as a result [9, 10]. Furthermore, vaccine responses are typically diminished in older individuals, resulting in lower antibody titers and reduced efficacy [11–14]. Multiple studies have established correlations between dysregulation in the adaptive immune system and negative clinical outcomes, but a detailed mechanistic understanding of how these changes in adaptive immune cell function affect responses to disease and/or vaccination is lacking. We focus our subsequent discussion on the relationship between observed differences in immune cell function and the pathology of infectious diseases that disproportionately affect the elderly, as well as research efforts in vaccinology that have been aimed at improving immune responses in older adults.

### Influenza virus

Influenza virus is one of the leading causes of respiratory infections among older adults. An estimated 50–70% of influenza-related hospitalizations impact adults over the age of 65 each year, with 70–90% of deaths related to influenza occurring among this same age group [115]. Numerous studies have reported influenza vaccine efficacy to be significantly lower in older adults compared to younger subjects, although there is some debate regarding how these statistics are reported [116, 117]. Nevertheless, the documented burden of influenza infection on the elderly population, combined with known deficiencies in vaccine-induced immune responses, emphasizes the need to understand the mechanisms governing adaptive immunosenescence as they relate to influenza-specific immune outcomes.

Humoral immunity is known to play an important role in preventing influenza virus transmission and infection, yet antibody responses have been shown to decline with age. A greater number of older adults fail to seroconvert (i.e., fourfold increase in post-vaccination antibody titer) relative to their younger counterparts, with seroconversion rates ranging from 10 to 30% in older adults compared to 50–75% in younger individuals [16, 118]. Recent evidence has suggested this decline in vaccine-induced humoral immunity is due to reduced neutralizing antibody production rather than a decline in total IgG output [21], and multiple studies have demonstrated that older adults fail to generate protective hemagglutination inhibition (HAI) antibody titers compared to younger adults following vaccination [16, 119]. Furthermore, older adults exhibit reduced diversity in their antibody repertoire following influenza vaccination, although a subset of elderly vaccine responders generate broadly cross-reactive antibodies that recognize multiple influenza strains [120]. The route of exposure may also be a key mediator of antibody responses, as differences have been observed between subjects naturally infected through the mucosal surfaces of the respiratory tract and those receiving intramuscular vaccinations [121]. Collectively, these findings are consistent with declining B cell function, but none have been mechanistically linked with negative clinical outcomes.

Cellular immunity is also strongly associated with protection against influenza, as some older adults have been shown to remain protected against infection even in the absence of robust antibody responses [122]. Declining T cell responses to influenza have been observed in both murine and human studies, suggesting age-related dysregulation in the T cell compartment may play a role in disease susceptibility. Studies have shown that older subjects experience altered frequencies of influenza-specific memory CD4^+^ T cell subsets post-vaccination relative to younger subjects [123], and these population shifts may alter the ability of memory T cells to effectively traffick to the lung in response to infection. Interestingly, studies in aged mice revealed that, although the total number of CD4^+^ T cells responding to infection in the lung did not differ from those in younger mice, there was a distinctive reduction in Th1 cells secreting inflammatory cytokines [124]. While deficiencies in CD4^+^ T cell responses could certainly impact the development of protective immunity, alterations in CD8^+^ T cells are equally important. Aged mice exhibit limited diversity in their TCR repertoire compared to younger mice, which decreases the magnitude of their response to the immunodominant influenza nucleoprotein epitope NP_366–374_ [125]. Although difficult to examine in humans due to the heterogeneity of responses [119], clonal expansion of CD45RA^+^CD28^−^CD8^+^ effector T cells has been implicated in causing Th1/Th2 cytokine imbalances that inhibit productive antibody responses [62]. Evidence from human studies also indicates that CD8^+^ T cell effector function is decreased following vaccination [126], although studies in mice indicate that decreased numbers of influenza-specific CD8^+^ T cells rather than functional changes are responsible for declining responses [127]. Collective differences between these reports suggest that CD4^+^ and CD8^+^ T cells may be differentially affected by immunosenescence, and further study is warranted in order to fully understand the effects of adaptive immunosenescence on cellular immunity to influenza.

Despite an incomplete mechanistic understanding, older adults are clearly at greater risk of influenza infection, and current evidence suggests this is due to dysregulation of the adaptive immune system. In an effort to improve clinical outcomes following influenza vaccination, vaccine formulations have been licensed specifically for use in the elderly. Fluzone® High-Dose, which contains 60 μg hemagglutinin per viral strain as opposed to 15 μg per strain in the standard dose vaccine, has been widely utilized in older adults and significantly increases humoral responses and efficacy [29]. More recently, the emulsion-based adjuvant MF59® has been added to influenza vaccines (Fluad™) and approved for use [28]. While the mechanisms of action are not completely understood, MF59® is believed to enhance innate immune responses and stimulate germinal center reactions [128, 129]. It should be noted that these two formulations have been globally employed to different degrees. Vaccines containing MF59® have been licensed for use in Europe since 1997 [130] but did not gain approval for use in the US until 2015. Alternatively, Fluzone® High-Dose has been licensed for use in the US since 2009 [29] but only received its first approval for use in Europe (UK) in 2019. Furthermore, these vaccines were not directly engineered to address any known mechanistic deficiencies in aging B cells; rather, these formulations advanced through clinical studies due to their overall improvement in vaccine efficacy. Despite their ability to generate higher protective antibody titers in the elderly, the mechanisms by which these two vaccines elicit such robust responses are poorly understood.

### Respiratory syncytial virus

Similar to influenza, respiratory syncytial virus (RSV) is another viral respiratory disease that has a disproportionate impact on older adults. Although RSV infection is usually mild in healthy young adults, serious complications can arise in older patients—particularly those with existing co-morbidities, such as chronic obstructive pulmonary disease and cardiovascular disease [131–134]. RSV infections often result in prolonged hospitalization and are associated with significant mortality rates (12–18%) in the elderly [135, 136]. Despite the impact of RSV on the aging population, the correlates of immunological protection remain poorly understood, although studies have indicated that adaptive immunosenescence may underlie the increased risk of severe disease among the elderly.

T cell responses have been shown to correlate with protection from RSV infection, and dysregulation of cellular immunity has been implicated in disease pathogenesis. Animal studies have shown that memory T cells mediate protection from disease and accelerate viral clearance but can contribute to tissue pathology if there is an imbalance in cytokine signaling [137–140]. Bias in cytokine production toward a Th2-type response has been noted to occur with age [67, 141], and studies have found that the number of circulating memory CD4^+^ and CD8^+^ T cells are also reduced in older adults [142, 143]. Specifically, RSV F protein-specific T cell responses were shown to be deficient in older adults compared to younger individuals [142]. These findings collectively suggest that cellular immunity may play a significant role in RSV pathogenesis, as both diminished and hyperactive responses can promote enhanced disease pathology in the elderly.

Although cellular immunity is predominantly associated with protection from RSV, correlations with humoral immune responses have also been demonstrated. Neutralizing antibody titers, as well as total IgG and mucosal IgA titers, have been associated with protection from RSV infection in adults [144–146]; however, some studies have reported equivalent neutralizing antibody titers between older and younger subjects, indicating cellular immunity as a stronger determinant of protection with age [140]. In experimental challenge models, mucosal IgA responses have been found to confer greater protection than serum neutralizing antibodies in adults, and deficits in IgA production have also been associated with an increased risk of recurrent RSV infection [147]. In contrast, one study has reported a stronger correlation between neutralizing antibody titer and disease susceptibility in the elderly [146]. While the precise role humoral immunity plays in protection from RSV remains unclear, it is apparent that age-related changes in both humoral and cellular immune responses may impact the progression of RSV infections in older adults.

Despite the apparent burden that RSV places on healthy aging, no vaccine is currently licensed for preventive use against RSV infection. This is an active area of research, as more than 60 vaccine candidates are currently in various stages of development; however, those few that have advanced to clinical testing have not demonstrated convincing efficacy [148]. The majority of vaccines in development for older adults are either nanoparticle or subunit-based formulations that primarily target the RSV F protein [148]. Clinical studies have failed to demonstrate improved vaccine efficacy despite showing robust immunogenicity, and insight into structural changes of the F protein may inform the design of new vaccine candidates. Studies have shown that vaccines using stabilized pre-fusion F protein generate higher neutralizing antibody titers compared to formulations containing post-fusion F protein [149, 150]. Furthermore, as described above, immunosenescence has been demonstrated to affect many of the factors directly associated with protection (memory T cell number and cytokine secretion patterns). This suggests that older adults may benefit from RSV vaccines specifically engineered to maximize immune responses despite immunosenescence – much like has been done with influenza. Consequently, research efforts to gain a fundamental understanding of RSV immunology in older adults are undoubtedly needed and will serve to inform the design of next-generation vaccines tailored for use in the elderly.

### Pneumococcal disease

Bacterial pneumonia is another common respiratory disease that is often deadly among older adults. Disease results from infection with *Streptococcus pneumoniae*, a common bacterial commensal that frequently (albeit transiently) colonizes the upper respiratory tract [151, 152]. While colonization is usually benign, migration or aspiration of *S. pneumonia* into the lower respiratory tract often results in pronounced disease progression [151, 152]. Mortality rates associated with pneumococcal disease range from 15 to 30% among the elderly [151], and with the increasing population of older adults, the number of hospital admissions related to pneumococcal pneumonia among adults > 65 years of age has been projected to increase by 87% [7]. Despite the growing disease burden, relatively few mechanistic studies of immunosenescence and pneumococcal immune responses have been conducted, although there has been significant progress made in the development of pneumococcal vaccines for older adults [153–155].

Humoral immunity is thought to play a key role in limiting the severity of pneumococcal disease, as deficiencies in either mucosal or systemic antibody production have been associated with poor medical outcomes [156, 157]. Serum IgG antibodies against *S. pneumoniae* have been identified as critical for preventing invasive bacteremia, while secretory IgA serves to mediate clearance of bacteria from the lung mucosa. Studies investigating the effects of aging on IgA responses in humans are scarce, but studies in mice have found IgA production following intranasal vaccination to be severely limited with age [158, 159]. Human studies have found that older adults (> 65 years of age) have significantly lower IgG antibody titers against many of the common pneumococcal serotypes compared to younger adults, suggesting that antibody titers wane over time [160–162]. Additionally, several studies have shown that antibodies from older adults have diminished opsonization activity against *S. pneumoniae* compared to those from younger adults, indicating there may also be functional deficiencies in antibody responses against pneumococcal antigens [17, 18].

While humoral immunity is primarily thought to mediate protection from disease, there are also important aspects of cellular immunity to consider. CD4^+^ T cells secreting IL-17 have been identified as key mediators of adaptive immune responses against *S. pneumoniae* [163], yet there are conflicting reports regarding age-related changes of T cell responses against pneumococcal infection. A study by Meyer and coworkers identified a significant increase in the percentage of CD4^+^ T cells in the lungs of older adults [164], while a separate study found no significant differences in the percentage of cytokine-secreting cells following stimulation with pneumococcal protein antigens [161]. Studies in mice have shown that CD4^+^ T cell responses can be generated by mucosal vaccination, but substantially more antigen is required to elicit responses in aged mice [159]. More studies are clearly needed in order to inform our understanding of mucosal immunology and aid the design of next generation vaccines against pneumococcal disease.

Two vaccine formulations have been currently licensed for clinical use against pneumococcal disease in older adults: a 23-valent carbohydrate vaccine (Pneumovax® 23) and a 13-valent glycoconjugate vaccine (Prevnar 13®) [165, 166]. Carbohydrate vaccines are poorly immunogenic as they do not inherently stimulate T cell responses, but Prevnar 13® overcomes this limitation via conjugation of the pneumococcal glycans to diphtheria toxoid [167]. In a randomized clinical trial, adults receiving the conjugate vaccine were found to suffer significantly fewer incidences of pneumococcal pneumonia (45% efficacy against non-invasive community-acquired pneumonia; ~ 75% efficacy against invasive pneumococcal disease) compared to subjects receiving a placebo [153]. Current evidence suggests an initial immunization with Prevnar 13® followed by subsequent immunizations with either vaccine provides the strongest antibody response [168], although there are still limitations to this approach. Serotypes excluded from the vaccine formulations can still lead to natural infections, leading to disease despite immunity against other serotypes. Further, comparative studies of the two vaccine formulations in older adults are lacking. Systems immunology studies comparing the responses to these two vaccines will serve to greatly increase our understanding of carbohydrate immunology and the immune response against pneumococcal disease.

### Herpes zoster

Although distinct from respiratory infections, herpes zoster (HZ) is another viral disease that manifests with age, and its pathology is strongly associated with declining cellular immunity. Nearly 100% of the adult population is exposed to varicella zoster virus (VZV) during their lifetime, which establishes latent infection within the dorsal root ganglia [169]. Reactivation of VZV infection is believed to be controlled by cellular immunity, but failure of the T cell compartment to maintain control of the infection with increasing age has been associated with the onset of HZ [12, 170]. This leads to an estimated 1 million cases of HZ in the United States annually that can severely impact the quality of life for older adults often due to complications with postherpetic neuralgia (PHN) [171]. Vaccine development efforts have proven effective in combating HZ in older adults, and efficacy studies have begun to simultaneously provide insight into the immunological mechanisms governing protection against HZ [172–174].

Two vaccine formulations have been licensed for clinical use against HZ, but the immunological responses between the two vaccines have been shown to differ significantly. The live attenuated zoster vaccine (Zostavax®) was the first to be approved for use in older adults, and early clinical studies demonstrated improved VZV-specific cellular immune responses despite limited efficacy (~ 51%) in adults over 60 years of age [175, 176]. Unfortunately, efficacy was found to significantly decline as age at the time of vaccination increased, decreasing to 41% in adults > 70 years of age and 18% in individuals > 80 years of age [175]. Protective immunity has also been shown to wane in the 6–8 years following receipt of the vaccine [12, 13]. As an alternative, a recombinant subunit vaccine (Shingrix™), consisting of recombinant glycoprotein E (gE) and the AS01_B_ adjuvant, was recently approved for use in older adults [177]. Following a two-dose schedule, the recombinant vaccine was found to have markedly improved efficacy (97%) in adults irrespective of age [172, 173]. Furthermore, protective immunity has been found to persist for close to a decade following the initial immunizations [30, 178]. The superior performance of the recombinant vaccine has been attributed to the development of robust gE-specific memory Th1-type responses, which are significantly lower in subjects receiving the live attenuated vaccine [174, 179]. Although the mechanisms governing these differences are not well understood, studies in mice have shown that the AS01_B_ adjuvant stimulates activation of antigen-presenting cells and macrophages in the draining LN [180].

Contributions from humoral immunity toward protection from HZ have been less well-defined, although a four-fold increase in VZV-specific antibody titers has been proposed as a correlate of protection for Zostavax® [181, 182]. Few studies have investigated specific aspects of humoral immunity against VZV, as evidence from the aforementioned clinical studies clearly implicates cellular immunity as a key determinant of disease protection. A recent study analyzing the functionality of antibodies following immunization with the live attenuated vaccine found that gE-specific memory B cell responses dominated, but these antibodies were unable to neutralize VZV in the absence of complement proteins or prevent the spread of the virus between cells in vitro [183]. These observations continue to suggest that antibody responses do not play a significant role in protective immunity against HZ, but studies investigating humoral immune response following administration of the recombinant vaccine are still lacking. Further studies of these two vaccines are warranted in order to fully elucidate the immunological mechanisms that govern durable protection against HZ.

## Future perspectives

### Study design considerations

There is no doubt that functional changes occur within the adaptive immune system during aging, but the need remains to better understand the mechanisms governing these adverse changes with age. Our current understanding is based upon findings suggesting that these changes are deleterious and correlate with negative clinical outcomes, yet it is important to understand the limitations of these studies when interpreting their results. Much of the work toward understanding the underlying mechanisms of adaptive immunosenescence has been carried out in rodent models, and discrepancies between rodent and human biology must be considered [184]. For example: 1) aging in mice occurs on a fundamentally different timescale; and 2) regulation of immune cell homeostasis is notably different from that in humans [44, 184]. Furthermore, immune cell subsets identified in mice may not perform the same function—or even exist—in humans; only recently have analogous T_VM_-like cell populations been identified [25]. Similarly, memory cell surface markers are known to differ between mice and humans; for example, CD27 is a well-established marker of memory B cells in humans, whereas murine memory cell populations lack expression of CD27 or an analogous phenotypic marker [185, 186]. A decline in murine DC function has been shown to occur with age [79, 80], but a similar change has not been observed in the human DC subsets studied to-date [77, 78]. Further studies of DC subsets are warranted in order to determine the full extent of age-associated dysregulation in human DCs, although there are technical and ethical limitations on this front. The rarity of certain DC subsets in circulation precludes isolation and mechanistic analyses, although advanced methodologies, such as single-cell RNA sequencing (scRNA-Seq) and mass cytometry (CyTOF), represent promising platforms that enable the analyses of rare cell populations in biological samples [187, 188]. Mechanistic studies of aging DC function should be carried out in humanized mouse models, as the isolation of tissue-resident DCs from human subjects remains too invasive for clinical implementation. While humanized mouse models are not perfect representations of human biology, they would allow for more detailed study and characterization of the biological mechanisms underlying immunosenescence in human DC subsets.

In addition to studying the mechanisms of aging, animal models are equally important for the study of disease and vaccine efficacy, yet limitations exist. Ferrets have been routinely used to evaluate influenza transmission, yet this model remains limited by continued variability in virus transmission as well as the significant costs and infrastructure necessary to house larger animal species [189]. The development of candidate vaccines for RSV is complicated by inconsistencies between rodent and primate models, as vaccine formulations that are highly effective in rodents often provide limited protection in chimpanzees and other primate species [190]. Studies of VZV outside of human hosts are even more limited, as there is currently no animal model that fully recapitulates disease pathogenesis for effective study [191]. Efforts have been made to establish safe and effective human challenge models for the study of influenza and RSV, but these studies have obviously remained limited to healthy young volunteers [192]. Despite these limitations, we must continue to rely upon carefully designed studies in animal models to investigate the molecular mechanisms of immunosenescence and disease, as ethical standards prohibit such invasive studies in high-risk human populations (e.g., older adults). Furthermore, we must review data from these studies critically, as fundamental differences in biology between the model species and humans may preclude further trials.

Studies that have addressed mechanistic questions in humans have done so largely in cells isolated from peripheral blood (PBMCs) and cultured in vitro, which are incomplete representations of the more complex biology occurring in vivo. In addition, alterations to cells in the periphery may not necessarily be characteristic of changes taking place within tissues and other isolated compartments of the immune system. Indeed, distinct aging profiles have been identified for immune cells in mucosal tissues [193], underscoring the importance of further investigations into mucosal immunology and aging. Another factor limiting our understanding of immunosenescence in humans is the paucity of longitudinal studies that have been conducted to date. Aside from some early studies evaluating immune risk profiles in Swedish octo- and nonagenarians [194, 195], the majority of studies have been cross-sectional by design. While these studies certainly allow for the identification of *differences* between young and old adult cohorts, they do not allow for the direct monitoring of sequential age-related *changes* within the same group of individuals. Longitudinal studies are inherently expensive due to their extended duration, and maintaining compliance and retention within a large cohort over the course of such a study is a daunting task. Despite these obstacles, longitudinal studies are on the rise [196–198] and may yield useful information regarding the aging immune system.

### Personalized vaccines for older adults

Despite the challenges facing research and development efforts, there is a critical need for personalized vaccines that can bolster immune responses in older adults and improve clinical outcomes [199–202]. In response to this demand for improved treatments among the aging population, vaccine formulations against influenza and HZ have been licensed for use despite an incomplete understanding of their mechanisms of action [28, 29, 177]. In order to develop the best possible treatments and advance our understanding of how adaptive immunosenescence truly affects vaccine response outcomes, a systems-level approach should be employed when evaluating vaccine responses. High-dimensional studies have proven informative in evaluating immunological responses due to the complex interconnectivity of the immune system [199, 203, 204]. Our own work in evaluating influenza vaccine responses in older adults has allowed for the identification of numerous immune signatures at the genetic and transcriptomic level that could inform on the mechanisms by which immunosenescence influences vaccine response outcomes. Transcriptomic analysis of PBMCs following influenza vaccination identified changes in the expression of several genes related to memory B cell responses and cellular metabolism [205], and further analysis of the transcriptome identified the enrichment of several genes (e.g., *SPON2*, *MATK*, *CST7*) that have not been previously associated with cellular or humoral vaccine responses [206]. Furthermore, integrated data analysis identified several epigenetic methylation signatures that were associated with the expression of genesets involved in humoral and cellular immune responses [207, 208]. The results from these studies highlight the potential for systems biology to identify and define the mechanisms underlying adaptive immunosenescence and to further understand how changes in the aging immune system might impact responses to infection and vaccination. Moreover, this type of mechanistic insight could allow for the rational design of adjuvants and/or vaccine formulations to address specific cells, pathways, or interactions that fail or are dysregulated during aging and result in sub-optimal adaptive immune responses.

Given the improvements in clinical efficacy that licensed vaccines for older adults have already demonstrated, it is imperative that we understand their mechanisms of action. Understanding how these vaccines and adjuvants are stimulating robust immune responses in the elderly may inform the rational design of new vaccine formulations for other diseases that exhibit similar biology. Our own research efforts are actively focused on deciphering transcriptomic differences between immune responses to the high-dose and MF59-adjuvanted influenza vaccines in a population of older adults, with the hopes of providing insight into the mechanism of action for these two vaccines [209]. Similar studies by others have also begun to identify immune response signatures that differentiate the recombinant and live attenuated HZ vaccines [174]. A combination of improved longitudinal study designs with high-dimensional systems analyses will likely allow for great strides to be made in addressing the impact of adaptive immunosenescence on vaccine responses in older adults.

## Conclusions

Although there are certainly distinct differences between the adaptive immune systems of older and younger adults, there is still much debate regarding how these changes impact responses to infection or vaccination. It is clear that at least some of these changes are detrimental, as evidenced by the decline in vaccine efficacy and increase in susceptibility to disease with age. In order to decipher the contributions of adaptive immunosenescence to these phenomena, systems-level analyses and longitudinal studies will be paramount. A more complete mechanistic understanding of changes in the immune system over time will provide essential insights into the biology of immunosenescence and greatly facilitate the rational design of personalized vaccines for older adults.

## Data Availability

Not applicable.
